# Probiotic performance of *B. subtilis* MS. 45 improves aquaculture of rainbow trout *Oncorhynchus mykiss* during acute hypoxia stress

**DOI:** 10.1038/s41598-024-54380-7

**Published:** 2024-02-14

**Authors:** Alireza Neissi, Hamed Majidi Zahed, Reza Roshan

**Affiliations:** Nuclear Agriculture Research School, Nuclear Science and Technology Research Institute, Karaj, 31465-1498 Iran

**Keywords:** Immunology, Microbiology, Molecular biology

## Abstract

The aim of this study was to produce mutant strains of *Bacillus subtilis* with high probiotic performance for use in the aquaculture of rainbow trout *Oncorhynchus mykiss*. The main strain of *B. subtilis* (MS) was irradiated with gamma rays (5.3 KGy). Subsequently, the *B. subtilis* mutant strain no. 45 (MS. 45) was selected for bacterial growth performance, resistance to acidic conditions, resistance to bile salts and antibacterial activity against *Aeromonas hydrophila* and *Pseudomonas fluorescens*. After 60 days, the rainbow trout (70.25 ± 3.89 g) fed with MS. 45 and MS were exposed to hypoxia stress (dissolved oxygen = 2 ppm). Subsequently, immune indices (lysozyme, bacterial activity and complement activity), hematological indices [hematocrit, hemoglobin, WBC, RBC, mean corpuscular volume (MCV)] and antioxidant factors (T-AOC, SOD and MDA)) were analyzed after and before hypoxia exposure. The expression of immunological genes (IFN-γ, TNF-α, IL-1β, IL-8) in the intestine and the expression of hypoxia-related genes (HIF-1α, HIF-2α, FIH1) in the liver were compared between the different groups under hypoxia and normoxia conditions. Growth, immunological and antioxidant indices improved in group MS. 45 compared to the other groups. Stress indices and associated immunologic and hypoxia expressions under hypoxia and normoxia conditions improved in MS. 45 compared to the other groups. This resulted in improved growth, immunity and stress responses in fish fed with the microbial supplement of MS. 45 (*P* < 0.05) under hypoxia and normoxia conditions, (*P* < 0.05), resulting in a significant improvement in trout aquaculture.

## Introduction

Studies support the use of beneficial probiotics and their corresponding effects on host health. Probiotics of the genus *Bacillus* spp. show considerable flexibility in synthesizing of antibiotic compounds and are considered natural factories of biologically active compounds^1^. These compounds of interest for biological control due to their potent antimicrobial activity, low toxicity and good biodegradability compared to their chemical counterparts^2^.

*Bacillus subtilis*, one of the probiotics of the genus *Bacillus* spp, has been shown to have beneficial effects on growth, strengthening the immune system, antimicrobial effects, increasing resistance to various diseases and reducing stress symptoms^3^. The probiotic properties of *B. subtilis* strains in aquatic animal cultures have been extensively studied^4–7^. The strain *B. subtilis* subsp. *spizizenii* ATCC 6633 has shown inhibitory effects on Gram-positive and Gram-negative enteropathogenesis in a laboratory model through the production of the bacteriocin subtilisin A. In addition, *B. subtilis* probiotics can adhere to intestinal cells to combat pathogens through a competitive elimination mechanism^8^. However, wild microbial strains produce only small amounts of antimicrobial compounds. This is because they need these compounds to compete and do not overproduce them. In addition, microorganisms have evolved regulatory systems that allow a strain to prevent excessive production of its metabolites.

Therefore, programs are needed to increase the production of these compounds for their commercial use^9^. Studies show that survival and growth rates increase by 8 to 34 percent when the right strain of these microorganisms is used^10^. These microorganisms can increase efficiency in combating pathogens in aquaculture^10^. Therefore, the creation of probiotic mutant strains with high efficiency in aquatic animals is of great importance.

The modification of mutagenesis in probiotic strains helps to improve the biological control possibilities or the synthesis of antibacterial metabolites. Chemical or physical mutagens (ultraviolet or gamma rays) are used for this purpose^11,12^.

*Aeromonas hydrophila* is a bacterium that belongs to the Vibrionaceae family and causes a variety of infections in fish^13–16^. This strain is an opportunistic pathogen responsible for *Aeromonas* motile septicemia in several fish species, including rainbow trout^17,18^. *Pseudomonas fluorescens* is a Gram-negative bacterium that grows optimally at 25–30 °C. It is found in soils, water bodies and water bodies. It is present and widespread in soil, water, plants and vegetation.

*P. fluorescens* is a common pathogen in aquaculture, affecting both invertebrates and vertebrates, particularly shrimp and fish^19,20^. Diseases caused by *P. fluorescens* have been observed in several farmed fish species, including turbot (*Scophthalmus maximus*), common carp (*Cyprinus carpio*), Nile tilapia (*Oreochromis niloticus*), Japanese flounder (*Paralichthys olivaceus*), and grass carp (*Ctenopharyngodon idella*)^21–23^. In previous studies, gamma irradiation has increased the secondary metabolites and antimicrobial capabilities of *B. subtilis*^12,24^. Considering that one of the most important components of the efficiency of a probiotic strain is the control of pathogens, investigating the efficacy of the mutant strain against common aquaculture pathogens such as *A. hydrophila* and *P. fluorescens* is of great value^25,26^.

Microbial supplementation could create better conditions for responding to stressful environmental conditions via the innate immune system. Hypoxia stress is a major stressor in aquaculture^27^. This type of stress affects the breeding and rearing of fish and can be mainly attributed to the following causes: Exposure to the aeration system, fish transportation, overpopulation and others. To our knowledge, there are no published data on the probiotic effects of the high-performing probiotic *B. subtilis* mutant substrain on rainbow trout (*O. mykiss*) under hypoxia conditions.

The aim of this study was to isolate the most peculiar mutant form of *B. subtilis* ATCC 6633 produced by gamma irradiation under in vitro conditions (growth pH, bile salt and antagonistic to pathogenic bacteria). The aim was to use the selected mutant strain for the aquaculture of trout under normoxic and hypoxic conditions.

## Results

### Growth stages of mutants and main strains

**Figure 1:**
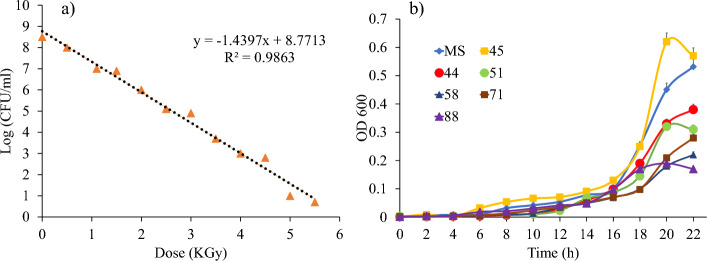
**(a)** Survival of *B. subtilis* during irradiation with doses of 1, 2, 3, 4 and 5 kGy. **(b)** Growth rate of the mutant strains and the main strain of *B. subtilis* for 20 h.

Using gamma radiation, a mutant strain with probiotic properties of *B. subtilis* bacteria was generated, and in the next steps the strains were selected and used for further experiments. The results of this study showed that the *B. subtilis* strain was completely inactivated with a dose of 6.09 KGy and 80% of the bacteria were inactivated with 4.93 KGy (Fig. 1a), so this dose was chosen as the effective dose (ED) for the generating of the mutant strains. The main strain was irradiated with the ED dose, and the bacterial colonies were collected and then analyzed for growth, resistance to bile acid, and pathogen resistance. The results showed that among the following mutant strains, even the growth rate of strain 45 was the highest (Fig. 1b). The lowest growth of the mutants was observed in strains 71 and 58.

### Bile salt tolerance

**Figure 2:**
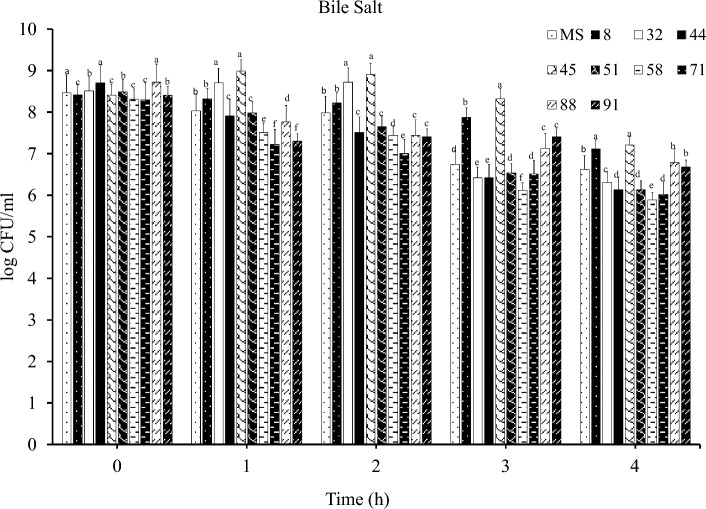
Average survival percentage of mutant and non-mutant strains of *B. subtilis* with 0.3% bile salt within 4 h.

The results showed that among the mutant and MS strains, strain 45 had the highest tolerance and strain 58 the lowest tolerance to bile salts (Fig. 2).

### Acid tolerance between mutant and non-mutant strains

The results showed that tolerance to simulated acidic conditions in the digestive tract differed between the *B. subtilis* mutant strains and the main strain (MS). The results showed that mutant strains 45 and 88 exhibited the highest tolerance to acidic conditions (pH 2 and 3) at all test time points, while strains 44 and 91 showed the lowest tolerance compared to the other strains (Table 1).

**Table 1:** Tolerance to acidic pH 2 and 3 mutant strains and main strain *B. subtilis* bacteria.

	Start	30 min	60 min	90 min	120 min
pH = 2					
MS	0.23 ± 8.47	0.35c ± 1.94	1.65 ± 0.63b	1.23 ± 0.23b	1.02 ± 0.21b
8	8.42 ± 0.12	1.82 ± 0.4c	1.53 ± 0.22b	1.37 ± 0.33b	0.63 ± 0.19c
32	8.51 ± 0.12	1.41 ± 0.32d	1.01 ± 0.23c	0	0
44	8.73 ± 0.79	1.48 ± 0.15d	1.09 ± 0.07c	0	0
45	8.72 ± 0.36	2.94 ± 0.23a	2.04 ± 0.42a	1.73 ± 0.33a	1.49 ± 0.23a
51	8.49 ± 0.45	0.8 ± 0.13e	0.63 ± 0.13d	0.41 ± 0.11d	0
58	8.31 ± 0.38	2.43 ± 0.25b	2.13 ± 0.41a	1.52 ± 0.18a	0.49 ± 0.11c
71	8.29 ± 0.44	1.82 ± 0.44c	1.61 ± 0.23b	1.11 ± 0.23c	0.62 ± 0.09c
88	8.41 ± 0.49	2.37 ± 0.22b	2.01 ± 0.47a	1.37 ± 0.27b	1.02 ± 0.39b
91	8.4 ± 0.33	0.75 ± 0.18e	0.49 ± 0.14d	0	0
pH = 3					
MS	8.4 ± 0.31	2.02 ± 0.38d	1.33 ± 0.26e	1.08 ± 0.36d	0.82 ± 0.23d
8	8.42 ± 0.12	3.12 ± 0.45b	2.59 ± 0.26c	2.27 ± 0.25c	1.92 ± 0.31b
32	8.51 ± 0.12	2.71 ± 0.55c	2.31 ± 0.44c	1.12 ± 0.42d	0.93 ± 0.17d
44	8.73 ± 0.79	2.78 ± 0.23c	2.45 ± 0.36c	1.34 ± 0.23d	0.87 ± 0.24d
45	8.72 ± 0.36	3.67 ± 0.31a	3.44 ± 0.29a	2.79 ± 0.18a	2.69 ± 0.23a
51	8.49 ± 0.45	2.21 ± 0.14	1.79 ± 0.35d	1.21 ± 0.31d	0.96 ± 0.55d
58	8.31 ± 0.38	3.2 ± 0.33b	2.92 ± 0.18b	2.32 ± 0.31b	2.08 ± 0.18b
71	8.29 ± 0.44	3.12 ± 0.41b	2.71 ± 0.23c	2.12 ± 0.28c	1.92 ± 0.18b
88	8.41 ± 0.49	3.41 ± 0.35a	3.11 ± 0.22b	2.55 ± 0.22b	1.79 ± 0.23c
91	8.4 ± 0.31	2.12 ± 0.48d	1.37 ± 0.32e	1.08 ± 0.36	0.82 ± 0.23d

Values indicate the mean ± standard deviation of three replicates. Values in a column with different superscripts are significantly different (*P* < 0.05).

### Antimicrobial activity of the mutant strains and the MS strain

**Figure 3:**
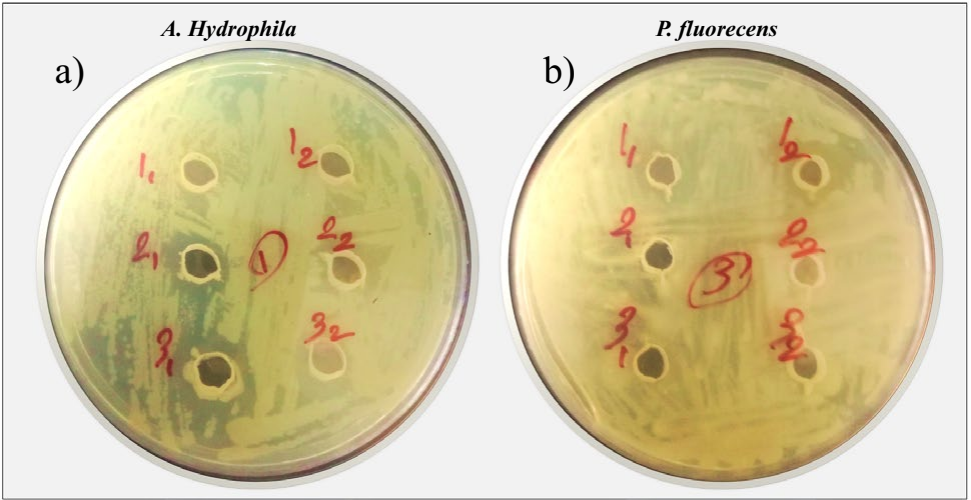
Antagonistic activities of *B. subtilis* mutant strains (1_1_, 1_2_, 3_1_, 3_2_) and the main strain (MS, 2_1_, 2_2_) against *A. hydrophila* and *P. fluorescens* by diffusion method in agar troughs.

The results showed that *B. subtilis* mutant strains No. 45 (MS. 45) and No. 51 exhibited the greatest antagonistic activity against *A. hydrophila* and *P. fluorescens*. Among the strains, strains No. 32 and No. 44 showed the lowest antagonistic activities compared to the other strains (Table 2, Fig. 3a,b).

**Table 2:** Antagonistic activities of *B. subtilis* mutant strains and the main strain (MS) against *A. hydrophila* and *P. fluorescens* by diffusion method in agar troughs.

	MS	8	32	44	45	51	58	71	88	91
*A. hydrophila (mm)*	17.2 ± 1.1^b^	17.8 ± 1.7^b^	14.1 ± 2.4^c^	16.9 ± 1.5^b^	28.2 ± 1.1^a^	22.4 ± 2.1^a^	17.1 ± 1.22^b^	16.5 ± 2.1^b^	15.2 ± 1.9^b^	16.1 ± 1.6^b^
*P. fluorescens (mm)*	14.4 ± 2.1^c^	15.1 ± 1.8^b^	13.9 ± 1.12^c^	14.2 ± 1.1^c^	25.7 ± 1.9^a^	2.4 ± .1.2^a^	16.5 ± 1.5^b^	14.4 ± 1.1^c^	15.5 ± 1.8^b^	16.2 ± 1.2^b^

The table shows the mean values ± standard deviation of three replicates. Values within a row with different superscripts are significantly different (*P* < 0.05).

### Growth of the rainbow trout

**Figure 4:**
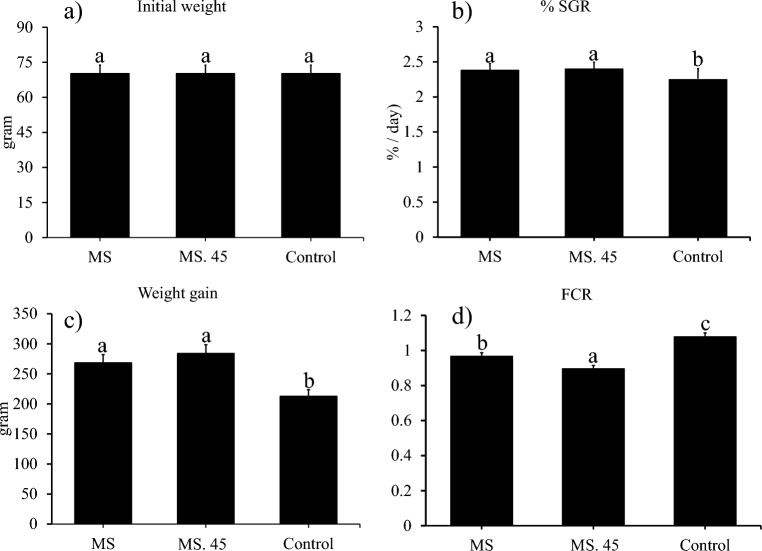
Comparison (mean ± SD) of initial weight **(a),** specific growth rate **(b),** weight gain **(c)** and feed conversion ratio (FCR) **(d)** in rainbow trout fed the B. subtilis main strain (MS), the mutant strain (MS. 45) and C (without microbial supplement) after 8 weeks of feeding (*P* < 0.05).

At the beginning of the rearing period, no significant difference was found between the weights of the different study groups (Fig. 4 a). Trout growth and SGR in MS. 45 and the MS group increased compared to the control group (Fig. 4b,c). The results also showed that the FCR rate in MS. 45 was lower than in the other groups, and the observed differences were statistically significant (Fig. 4 d).

### Hematologic indices under hypoxia and normoxia conditions

The result showed that under hypoxia and normoxia conditions, hematocrit, hemoglobin, WBC (× 10^3^/µI), RBC (× 10^6^/µI) MS and MS. 45 groups showed higher values than the control group. But %MCHC in MS and MS. 45 lower than in the control group (Table 3). Hematocrit, hemoglobin, WBC (× 10^3^/µI), RBC (× 10^6^/µI) increased under hypoxia conditions compared to normoxia conditions.

**Table 3:** Hematological indices hematocrit, hemoglobin, leukocytes, erythrocytes (mean corpuscular volume (MCV), mean corpuscular hemoglobin concentration (MCHC)) in rainbow trout sera fed with *B. subtilis* main strain (MS) and mutant no. 45 (MS. 45) under hypoxia and normoxia conditions.

	%Hematocrit	Hemoglobin	WBC (× 10^3^/µI)	RBC (× 10^6^/µI)	%MCHC	MCV (fl)
Hypoxia Condition						
MS	41.52 ± 1.12^a^	10.31 ± 2.13^a^	7.36 ± 0.45^a^	1.25 ± 0.02^b^	49.6 ± 4.5^a^	332 ± 33.5^a^
MS. 45	42.59 ± 1.28^a^	11.02 ± 0.59^a^	7.41 ± 1.12^a^	1.26 ± 0.03^b^	51.7 ± 3.8^a^	338 ± 34.6^a^
C	37.41 ± 2.12^b^	9.49 ± 0.78^b^	7.32 ± 0.99^b^	1.32 ± 0.04^a^	50.7 ± 2.9^a^	283 ± 41.2^b^
Normoxia condition						
MS	37.54 ± 3.56^b^	9.51 ± 1.54^a^	7.40 ± 0.87^a^	1.22 ± 0.08^b^	50.6 ± 4.1^a^	307 ± 44.5^a^
MS. 45	38.25 ± 3.89^b^	10.25 ± 0.36^a^	7.45 ± 1.02^a^	1.21 ± 0.11^b^	53.5 ± 3.8^a^	316 ± 33.6^a^
C	35.36 ± 2.99^c^	8.12 ± 0.36^b^	7.38 ± 0.78^b^	1.36 ± 0.13^a^	45.9 ± 3.9^b^	260 ± 29.1^b^

The table shows the means ± SD of three experimental replicates. Values within the same columns with different superscripts differ significantly (*P* < 0.05).

### Complete blood count (CBC), proteins and immunological indices under hypoxia and normoxia conditions

We investigated the complete blood count (CBC) and immunological indices of trout in the MS. 45 fed group compared to the control group under hypoxia and normoxia conditions. The results showed that the neutrophils and monocytes in the MS and MS. 45 groups were higher than the control group at the end of the experiment in both hypoxia and normoxia.

**Figure 5:**
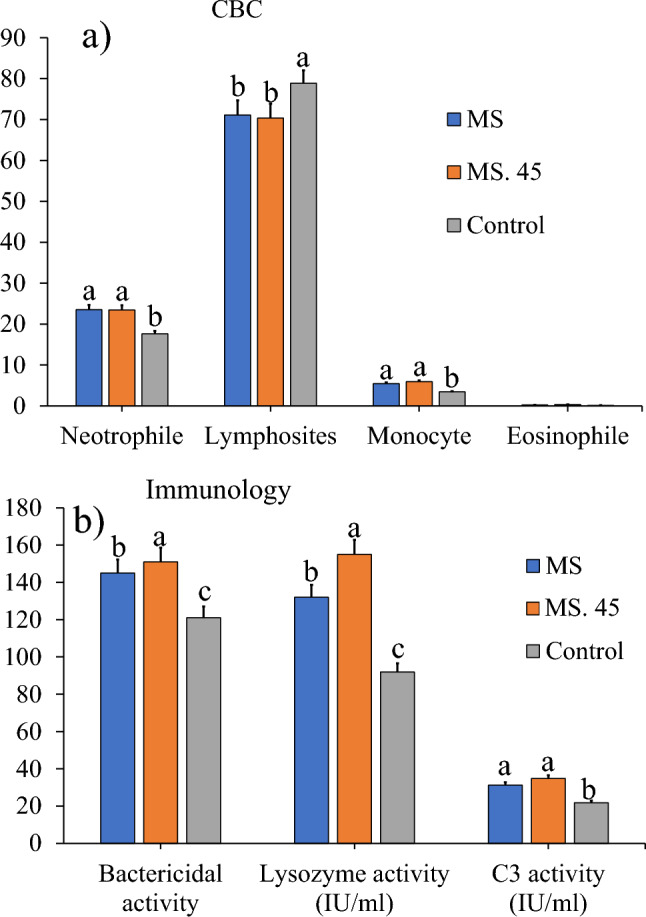
Comparison (mean ± SD) of **(a)** complete blood count (CBC), neutrophil lymphocytes, monocytes, eosinophils; and **(b)** immunological performance; bacterial activity, lysozyme, C3 activity, in rainbow trout sera treated with *B. subtilis* main strain (MS), *B. subtilis* Mutant strain number 45 (MS. 45) and control (no microbial supplementation) after 8 weeks feeding (*P* < 0.05).

The lymphocytes in the MS and MS. 45 groups were lower at the end of the experiment (Fig. 5 a) under hypoxia and normoxia conditions than in the control group. The result showed that the bacterial activity, lysozyme and C3 activity in the MS. 45 group were higher under both hypoxia and normoxia conditions than in the control and MS groups (Fig. 5 b).

The results showed that total protein, globulin and albumin in the sera of rainbow trout from the MS and MS. 45 groups compared to the control group under hypoxia and normoxia conditions (Table 4).

**Table 4:** Total protein, globulin, and albumin in sera of rainbow trout fed the main strain (MS) of *B. subtilis* and *B. subtilis* MS. 45 under hypoxia and normoxia conditions.

Hypoxia condition	Total protein	Globulin	Albumin
MS-Hypoxia	4.26 ± 0.08^a^	1.69 ± 0.12^b^	2.57 ± 0.65^b^
MS. 45-Hypoxia	4.65 ± 0.12^a^	1.87 ± 0.22^a^	2.78 ± 0.74^a^
Control-Hypoxia	3.56 ± 0.21^b^	1.43 ± 0.45^c^	2.13 ± 0.65^c^

Normoxia condition	Total protein	Globulin	Albumin
MS-Normoxia	4.56 ± 0.33^b^	1.79 ± 0.65^b^	2.77 ± 0.54^b^
MS. 45-Normoxia	4.85 ± 0.28^a^	1.91 ± 0.32^a^	2.94 ± 0.35^a^
Control-Normoxia	3.88 ± 0.41^c^	1.56 ± 0.45^c^	2.32 ± 0.61^c^

The table shows the mean values ± SD of three experimental replicates. Values within the same columns with different superscripts differ significantly (*P* < 0.05).

### Stress indicators under hypoxia and normoxia conditions

**Figure 6:**
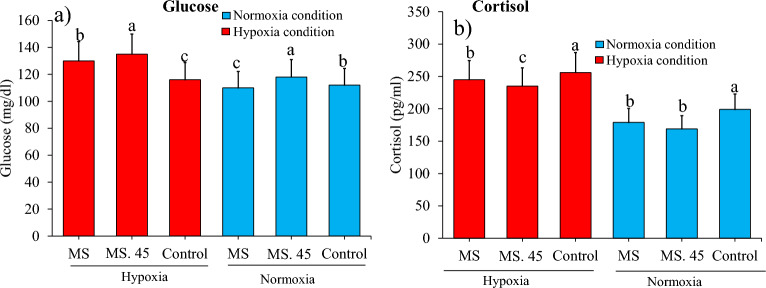
**(a)** comparison (mean ± SD) of glucose and **(b)** cortisol in sera of rainbow trout treated with *B. subtilis* main strain (MS), MS. 45 (*B. subtilis* No. 45) and the control (no microbial supplementation) after 8 weeks (*P* < 0.05).

Cortisol and glucose increased under hypoxia conditions compared to normoxia conditions in all groups. The result showed that cortisol was lower in MS 45 and MS under hypoxia and normoxia conditions. The result showed that glucose increased under hypoxia conditions in all groups (Fig. 6).

### Antioxidant-related indicators under hypoxia and normoxia conditions

**Figure 7:**
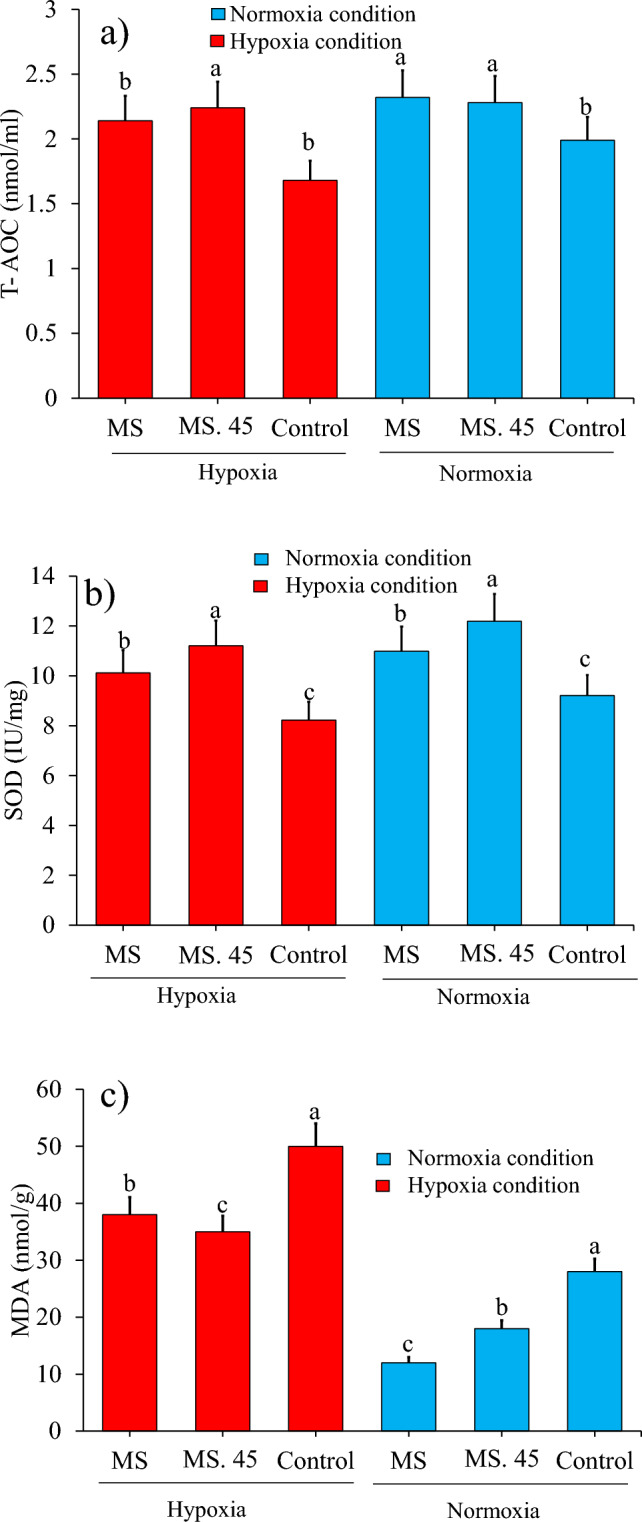
Comparison (mean ± SD) of antioxidant factors (a). T-AOC, (b) SOD and (c) MDA in rainbow trout treated with B. subtilis main strain (MS), B. subtilis No. 45 (MS. 45) and control (no microbial supplementation) after 8 weeks feeding (*P* < 0.05).

Although T-AOC and SOD decreased under hypoxia conditions, the results showed that the indices in the sera of rainbow trout fed with MS and MS. 45 were higher than in the control group after 8 weeks under hypoxia and normoxia conditions (Fig. 7a,b). We also observed opposite conditions for the amount of MDA in the hypoxia and normoxia groups of MS and MS. 45 (Fig. 7c).

### Quantitative PCR (q-PCR) under hypoxia and normoxia conditions

**Figure 8:**
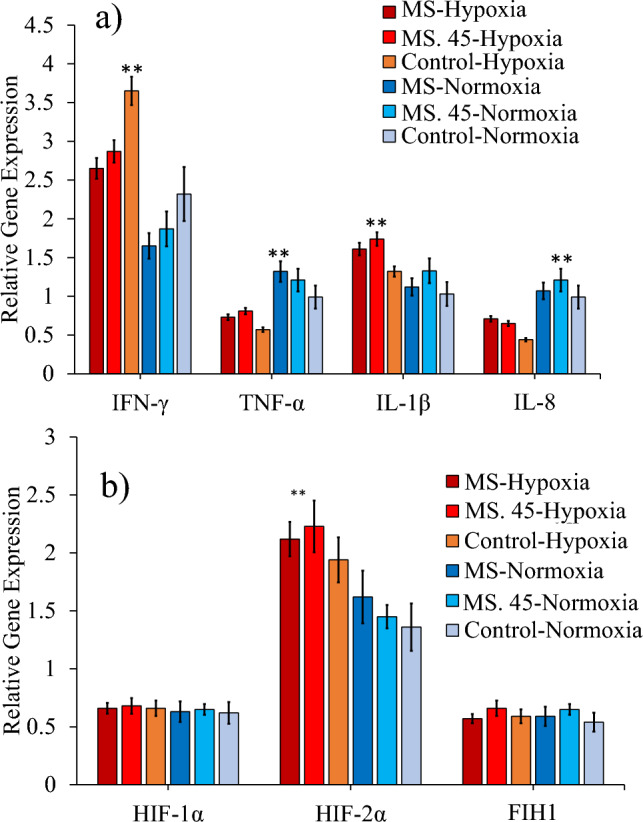
Comparison (mean ± SD) of the expression of **(a).** immunological genes (IFN-γ, TNF-α, IL-1β, IL-8) in the intestine and the expression of **(b)** hypoxia- related genes (HIF-1α, HIF-2α, FIH1) in the liver of rainbow trout fed with the *B. subtilis* main strain (MS) and mutant No. 45 (MS. 45) after 8 weeks under hypoxia and normoxia conditions. MS (main strain), control (no microbial supplementation), and No. 45 (*B. subtilis* No. 45) (*P* < 0.05).

The results showed that the immunological gene expression of IFN-γ was increased in the control group under hypoxia and normoxia conditions (Fig. 8a). The expression of IL-β was increased in all groups under hypoxia conditions, but the expression was higher in the MS 45 and MS groups. The expression of TNF-α and IL-8 decreased under hypoxia stress in all groups, but the expression in the B. subtilis MS and MS. 45, however, was higher under hypoxia and normoxia conditions than in the control group (Fig. 8a). A comparison of the immunological genes of rainbow trout fed *B. subtilis* MS and MS. 45 fed rainbow trout showed that the expression of IFN-γ and IL-1β was increased in all groups, but gene expression in the control group was higher than in the other groups under hypoxia and normoxia conditions (Fig. 8a). The expression of TNF-α and IL-8 decreased under hypoxia stress, but the expression in *B. subtilis* MS and MS. 45 was higher under hypoxia and normoxia conditions (Fig. 8a). Comparison of the expression of hypoxia-associated genes HIF-1 and α FIH1 showed that the genes in the liver of rainbow trout fed the *B. subtilis* main strain (MS) and MS. 45 did not differ from the control group under hypoxia and normoxia conditions (Fig. 8b). However, HIF-2α expression increased in all groups under hypoxia stress, with the increase being highest in group no. 45 (Fig. 8b).

## Discussion

To increase the efficacy of probiotic strains, methods such as the creation of mutant strains, genetic manipulation, and environmental adaptation can be used^28,29^. One of the methods used to create mutations is the use of radiation in the form of ionizing and non-ionizing rays. Gamma radiation, which belongs to ionizing radiation, can play an important role in mutation due to its high transmittance and ability to affect the genome^29,30^. Thus, by using gamma radiation and creating mutant strains and screening them carefully, a highly effective probiotic strain can be obtained. In a study with mutant strains of *L. plantarum*, it was found that a certain biochemical property of the cell surface can enhance its probiotic effect^31^. Similar results were observed with *Lactobacillus acidophilus* strains with lipoteichoic deficiency and *Lactobacillus rhamnosus*^32,33^.

In the current study, gamma ray mutation was performed to enhance the probiotic properties of *B. subtilis* ATCC 6633 bacteria under gamma radiation. The results showed that *B. subtilis* MS. 45 was able to maintain its viable state under laboratory conditions against bile salts and acidic conditions better than the original strain.

Probiotics are microorganisms that improve the microbial balance in the intestines of humans and animals when consumed in sufficient quantities (10^8^–10^12^ CFU per day, depending on the type of bacteria). *Bacillus* probiotics and their metabolites are promising candidates for biotechnological applications and fermented foods^34^. Compared to common probiotic species such as *Lactobacillus* and *Bifidobacterium*, the bacilli are extremely resistant to heat and chemicals and can maintain the extreme pH of gastric fluid by forming spores, making them ideal food additives for humans and animals^35^. Other beneficial effects of *B. subtilis* include the production of enzymes, amino acids, and antibiotic compounds such as bacteriocins, as well as the strengthening of gut-associated lymphoid tissue (GALT) in humans and animals^8,36^.

Previously, *B. subtilis* ATCC 6633 was shown to have an inhibitory effect on Gram-positive and Gram-negative enteropathogenic pathogens by producing the bacteriocin subtilosin A in an in vitro model. In addition, *B. subtilis* probiotics can adhere to intestinal cells to combat pathogens through a competitive elimination mechanism^8^. According to previous reports, antibiotic peptides containing subtilin and subtilosin A produced by *B. subtilis* ATCC 6633 can inactivate a large number of Gram-negative and especially Gram-positive bacteria by creating voltage-dependent pores in the cytoplasmic membrane of the bacteria. In addition, protease and pronase enzymes produced by *B. subtilis* are known to inhibit the action of CPE, and such proteolytic enzymes are likely to be present in our extract. Furthermore, a non-specific steric barrier mechanism of probiotics seems to block the binding of pathogens to their cellular receptors, which is probably also the case for CPE^37,38^.

A previous study has shown that strain *B. subtilis* ATCC 6633 releases factors against *C. perfringens, A. hydrophila*, and *P. fluorescens*, a causative agent of necrotizing enteritis in aquatic animals^39,40^. In the case of *B. subtilis* strains LFB112 and 8A isolated from plants and soil were also found to have an inhibitory effect against *C. perfringens* through the production of bacteriocins^41^. In one study, the antagonistic activity of strain ATCC 6633 against *A. hydrophila* and *P. fluorescens* was also demonstrated^39^.

The use of probiotics in the diet of aquatic animals can have a positive effect on growth performance, feed consumption, and physiological condition^42^. This increase in nutrient digestibility is due to the increased availability of nutrients resulting from the introduction of probiotics in aquatic animals.

Many studies have shown that the secretion of digestive enzymes in sea bass (*Centropristis striata*) and rohu (*Labeo rohita*) was improved after feeding with probiotics^43^. In addition, several fish species fed with diets containing *B. cereus*, *B. subtilis, B. licheniformis* and *E. faecium* showed higher feed conversion and growth^44,45^. In the second part of this study, the results showed that trout fed with MS. 45 showed significantly better feed conversion and higher growth than the group fed control feed and even the original strain^46^. Probiotics, especially the genus *Bacillus*, improve digestive activity due to their ability to produce exoenzymes. They can improve nutritional efficiency by stimulating digestive enzymes, leading to better absorption and greater animal growth. Studies on feeds containing probiotics show the potential role of these probiotics in improving the balance of intestinal microflora and the production of extracellular enzymes to increase feed efficiency^6,43,45^.

Environmental stress, especially stress, usually suppresses the immune system of aquatic animals, which, if persistent, can lead to physical reactions and even death. Based on the above definitions, aquatic animals are usually exposed to various stress factors, such as transportation, malnutrition, stocking density, rearing temperature, anoxia, hypoxia, etc.

Environmental stress in aquatic animals can strongly influence physiology and oxidative stress. By improving the antioxidant status of the diet, probiotics help in the uptake of antioxidants that can act as a defense mechanism in the gut microbiome to overcome stress. The protective mechanism of aquatic animals has been reported to be influenced by the natural gut microflora, usually consumed in the form of probiotics, which enhance immunity, especially the protective HSP under stressful conditions.

In this study, hypoxic shock, hematologic indicators, immunologic stress and the expression of genes involved in hypoxic stress and immunity were investigated. The results showed that stress leads to an increase in glucose and cortisol, but not to the same extent in all groups and especially improved in the MS. 45. Hematologic indicators, including white and red blood cell counts, hematocrit and hemoglobin, were all affected by the role of the probiotic.

Probiotic supplements to reduce the harmful effects of various stressors in aquaculture, when mixed with vitamins and nutrients, can lower levels of biochemical stress and lead to a reduction in cortisol levels. Scientific research shows that probiotics lead to an improved immune response through the interaction between the bacterial cells and the host's intestinal epithelial cells that control intestinal immunity. In addition, probiotics can stimulate the body's immune system by creating metabolic pathways against infectious diseases, and this immune stimulation can have a positive effect on cellular immune function^6,42^.

In the current experiment, the results showed that, on the one hand, the immune indicators showed that the number of neutrophils increased and that of lymphocytes decreased in CBC group MS. 45 under both hypoxia and normoxia conditions. On the other hand, the activity of lysozyme, bacterial activity and complement were increased in both groups of the main *Bacillus* strain and especially in group MS. 45 under both hypoxia and normoxia conditions.

## Conclusions

In the present study, generation of the mutant strain *B. subtilis* MS. 45 by gamma irradiation increased the antagonistic activity against the important pathogenic bacteria in fish farming *A. hydrophila* and *P. fluorescens* as well as the growth and production of biomass under in vitro conditions. According to the in vivo results of this study, growth, immunity and stress responses improved in the fish treated with the microbial supplement of MS. 45 (*P* < 0.05) under hypoxia and normoxia conditions, (*P* < 0.05), resulting in a significant improvement in trout aquaculture.

## Materials and methods

### Preparation of the *B. subtilis* strain

The freeze-dried form of the main bacterial strain *B. subtilis* subsp*. spizizenii* ATCC 6633 was prepared from the microorganism bank of the National Center for Genetic and Biological Resources of Iran with the accession number IBRC-M 10,808. In the next step, the bacteria were serially diluted up to eightfold in 0.9% NaCl saline to measure primary activation and transferred to 8 cm plates of tryptic soy agar (TSA) culture medium. In the next step, the plates were transferred to a 30 °C incubator. After one night of incubation and checking the growth of each colony, the corresponding plates were transferred to the refrigerator.

### Generation of mutant strains

A fresh *B. subtilis* colony was transferred to a test tube containing 5 ml of TSB broth and, after overnight incubation at 30 °C, transferred to 500 ml flasks containing 100 ml of autoclaved TSB broth in a 30 °C incubator and then removed during the logarithmic growth phase. In the next step, the cells were collected by centrifugation at 8,000 rpm for 10 min and washed twice with sterilized 0.65% NaCl solution.

The suspension was then prepared with 0.65% NaCl solution and irradiated in the next phase with doses of 1, 2, 3, 4 and 5 KGy from the Canadian Gamacell device Nordian 220 with a dose of 0.92 Gy/sec from Co-60. After irradiation, the bacteria were serially diluted 8–tenfold in 0.65% saline, plated on TSA and incubated overnight at 30 ℃. The number of colonies was counted and the effective mutagenesis dose (5.3 KGy) was determined based on the 80% mortality rate. The main strain prepared in 0.65% saline was irradiated with a dose of 5.3 KGy and then transferred to TSA media for one night. The potentially probiotic colonies were isolated and analyzed separately for growth, bacterial bile salt tolerance, bacterial acid tolerance and antimicrobial activity against *A. hydrophila* and *P. fluorescens.*

### Bacterial growth

To obtain bacterial growth stages, a single colony of the selected strains and the main strain was transferred overnight to test tubes containing 5 ml TSB and then incubated overnight in 100 ml flasks containing 50 ml TSB at 30 °C and 140 rpm. Since the start of inoculation of the bacteria to the flasks, 1,000 µl of the bacterial cultures were removed with a pipette 0, 2, 4, 6, 8, 12, 14, 16, 18 and 20 h after inoculation and transferred to cuvettes to determine the OD600 with a spectrophotometer (*n* = 3).

### Bacterial bile salt tolerance

A single colony of selected unirradiated and irradiated *B. subtilis* substrains was transferred overnight to test tubes containing 5 ml TSB and then transferred overnight at 30 °C to 100 ml of TSB. The bacterial cells were then separated at 5,000 rpm for 20 min at 4 °C. The pellets were washed twice with PBS and then resuspended in TSB containing 0.3% bile salts. The bacterial suspensions were incubated at 30 °C for 0, 1, 2, 3 and 4 h. Viable cells were counted by plating tenfold dilutions of the culture in PBS on TSA and incubating overnight at 30 °C^47^.

### Bacterial acid tolerance

For the acid tolerance test, the pH in TSB medium with normal HCL 1 M was adjusted to 2 and 3. Then the selected unirradiated and irradiated *B. subtilis* substrains were inoculated onto the TSB media (pH 2 and 3) and incubated overnight at 30 °C (*n* = 3); then the cultures were diluted and the number of colonies (pH 2 and 3) on TSA medium was counted. Resistance to acidic conditions was determined by the survival value of the colonies.

### Antimicrobial activity against *A. hydrophila* and *P. fluorescens*

The antagonistic activity of unirradiated and irradiated *B. subtilis* substrains against the pathogens *A. hydrophila* and *P. fluorescens* was determined using the agar well diffusion method. 100 µl of the cell-free supernatant of the unirradiated and irradiated strains were inoculated into a 7 mm diameter well in TSA containing the test pathogens *A. hydrophila* and *P. fluorescens* for overnight incubation (*n* = 3)^28,48^. The diameter of the inhibitory zone was determined after 24 h of incubation.

### Preparation of the lyophilised form of bacteria of different strains

The selected *B. subtilis* bacterial strain number 45 (see Results section) and the non-irradiated strain were freeze-dried for the next step of fish feeding. Individual colonies of bacterial strain number 45 and the non-irradiated strain were incubated overnight at 30 ℃, 2,000 ml TSB. Then the contents of the media were transferred to sterile 50 ml tubes and centrifuged at 6,000 rpm for 15 min. The bacterial cells were mixed evenly with 10% dry milk powder and then placed in a − 70 freezer. The 50 ml tubes be placed in the freeze dryer. At the end, the concentration of bacteria per gram of freeze-dried powder (CFU/g) was calculated on TSA for the mutated and the non-irradiated groups.

### Animal experiments

Animal experiments were performed in accordance with the protocols approved by the Ethics Committee of the Faculty of Science, University of Tehran and in accordance with the relevant guidelines and regulations (protocol number: E52# 412–15 and ARRIVE guidelines).

Healthy and disease-free rainbow trout with an average weight of 70.25 ± 3.89 g were obtained from a farm in Karaj. 225 trout were distributed in nine 300-L fiberglass tanks with 250 L of water at 14.2 ± 1.8 °C. In this study, 3 experimental groups were considered: Control, MS and MS. 45. In the control group (C), the feed was coated with 1% fish oil and no bacteria were added. 10^9^ CFU of lyophilized powder of *B. subtilis* sub-strain No. 45 and 10^9^ CFU/g of lyophilized powder of the main strain were added to the MS. 45 and MS groups and coated with 1% fish oil as a carrier. The fish were fed 3% of body weight twice daily for two months (CP = 37.9 ± 3.1), divided into three feedings. The growth indicators for rainbow trout consisting of initial weight, percentage weight gain, SGR (% day-1) and FCR in groups C, MS. 45 and MS after 60 days were calculated.

### Hematologic, immunologic, stress and antioxidant indices under hypoxia and normoxia conditions

First, 3 normal trout from the experimental groups (MS, MS. 45 and control) were sampled under normoxic conditions. The aeration and water flow in the tanks were stopped and the volume of water in the tanks was reduced to 50 L to create a hypoxic environment. DO was continuously monitored using a DO meter (Eutech 300, Singapore).

When the DO level in the tank had dropped to 2 mg/L^49^ after 6 min, three fish from each experimental unit (MS, MS. 45 and control) were caught at random. First, they were anesthetized with clove powder^50,51^. Each fish was dried with a clean towel, and blood samples were collected from the tail using a 5-ml syringe. Each blood sample was immediately transferred to 1.5-ml tubes. Hematologicfactors such as red and white blood cell counts were determined according to Harikrishnan et al. and Houston et al.^52^; hemoglobin concentration and hematocrit, MSV, MCHC Complete blood count (CBC), was measured according to Drabkin and David^53^; total protein was measured according to Nonaka^54^, bacterial activity was examined according to Budiño, Cal, Piazzon and Lamas^55^, lysozyme and complement activity (C3) were measured according to Yano^56^, albumin and total globulin according to Siwicki and Anderson^57^ were measured under hypoxia and normoxia conditions. Cortisol and glucose levels were determined in a certified laboratory using a biochemical analyzer. Serum MDA, SOD, and T-AOC levels were determined using a commercial kit (Man Company) according to the manufacturer's methods under hypoxia and normoxia conditions.

### Expression of immunological and hypoxia-related genes under normoxia and hypoxia conditions

In addition, kidney and intestinal samples were taken after dissection from all fish of the different groups under hypoxia (MS, MS. 45 and control) and normoxia conditions (MS, MS. 45 and control). To extract the RNA, the samples were first homogenized in liquid nitrogen using a mortar and pestle and then kept at room temperature for 5 min. Then, 700 µl of RLT buffer (each milliliter of RLT buffer contained 10 µl of β-mercaptoethanol) was added to each of the samples. In the next step, the collected samples were transferred to tubes containing 0.1 mm beads from Sigma Aldrich, USA.

In this step, the cell walls of the samples were disrupted using the cooled Precellys® tissue lyser. The RNA extraction kit RNeasy Mini Kit from Qiagen, Germany, was used for the extraction according to the manufacturer's protocol. To remove DNA, samples were treated with DNase (DNase 25 µl + 175 µl RDD buffer) for 15 min at room temperature. The extracted RNA was transferred to a nanodrop on ice and the RNA concentration was measured.

For cDNA synthesis, the gDNA buffer was first incubated at 37 °C for 1 min. For each sample, 2 µl of this buffer and 12 µL of RNA were added at a concentration of 1,000 ng/µL. The samples were subjected to PCR at 42 °C for 2 min and then placed on ice for 2 min. In this step, 4 µl of RT buffer, 1 µl of RT, and 1 µl of oligo primer were added to each of the samples. Subsequently, the cDNA of the prepared samples was synthesized with a thermal cycler for 15 min at 42 degrees and 3 min at 95 °C.

The cDNA concentration was measured using Nanodrop, and samples were prepared for reading and measurement of gene expression using the QIAGEN OneStep RT-PCR kit. For each experimental unit, 3 replicates were considered. Each replicate contained 10 µl of SYBer green master mix, 2 µl of cDNA, 7 µl of RNase-free water, 0.5 µl of primer R and 0.5 µl of primer. The prepared primers (Supplementary Table 1) and the expression of IFN-γ, TNF-α, IL-1β, IL-8 (immunologically related gene) in the intestine and the expression of hypoxia-related genes (HIF-1α, HIF-2α, FIH1) were measured by q-PCR according to Rahimi et al. 2020^58^. Relative gene expression levels were determined using the Agilent Technologies Strata Gene Mx3,0005P and calculated according to the manufacturer’s instructions^59^. Gene expression was analyzed^58^.

### Ethics approval

We confirm that all experimental research and field studies on fish, including the collection of samples, were in accordance with relevant institutional, national (Ethics Committee of the Faculty of Science, University of Tehran Protocol Number = E52# 412–15) and international guidelines and laws. All material is the property of the authors and/or no permissions are required.

### Statistical analysis

The normality of the data was tested using the Kolmogorov–Smirnov test. One-way ANOVA analysis of variance was performed to compare the data means. The significance level between treatments was determined using the Tukey test at the 5% level. The statistical analysis was performed using SPSS 19 software in the Windows environment.

### Supplementary Information

Supplementary Table 1.

## Data Availability

Some or all of the data in this study are available upon reasonable request to the corresponding author.

## References

[1] Merrifield DL (2010). The current status and future focus of probiotic and prebiotic applications for salmonids. Aquaculture.

[2] Bóka B (2016). Ion trap mass spectrometry of surfactins produced by *Bacillus subtilis* SZMC 6179J reveals novel fragmentation features of cyclic lipopeptides. Rapid Commun. Mass Spectrom..

[3] Tegegne, B. A. & Kebede, B. Probiotics, their prophylactic and therapeutic applications in human health development: A review of the literature. *Heliyon*, e09725 (2022). 10.1016/j.heliyon.2022.e09725 PMC9240980 35785237

[4] Olmos J, Acosta M, Mendoza G, Pitones V (2020). *Bacillus subtilis*, an ideal probiotic bacterium to shrimp and fish aquaculture that increase feed digestibility, prevent microbial diseases, and avoid water pollution. Arch. Microbiol..

[5] Chen H, Ullah J, Jia J (2017). Progress in *Bacillus subtilis* spore surface display technology towards environment, vaccine development, and biocatalysis. Microb. Physiol..

[6] Liu H (2017). Dietary administration of *Bacillus subtilis* HAINUP40 enhances growth, digestive enzyme activities, innate immune responses and disease resistance of tilapia. Oreochromis Niloticus. Fish Shellfish Immunol..

[7] Nawaz A, Irshad S, Hoseinifar SH, Xiong H (2018). The functionality of prebiotics as immunostimulant: Evidences from trials on terrestrial and aquatic animals. Fish Shellfish Immunol..

[8] Ye X, Li P, Yu Q, Yang Q (2013). *Bacillus subtilis* inhibition of enterotoxic Escherichia coli-induced activation of MAPK signaling pathways in Caco-2 cells. Ann. Microbiol..

[9] Pham JV (2019). A review of the microbial production of bioactive natural products and biologics. Front. Microbiol..

[10] Martínez Cruz, P., Ibáñez, A. L., Monroy Hermosillo, O. A. & Ramírez Saad, H. C. Use of probiotics in aquaculture. *Int. Sch. Res. Notices* **2012** (2012). 10.5402/2012/916845 PMC3671701 23762761

[11] Haggag WM, Mohamed H (2007). Biotechnological aspects of microorganisms used in plant biological control. Am. Eurasian J. Sustain. Agric..

[12] Manikandan A (2022). Gamma-induced mutants of *Bacillus* and Streptomyces display enhanced antagonistic activities and suppression of the root rot and wilt diseases in pulses. Biomol. Concepts.

[13] Dong J (2021). Resveratrol influences the pathogenesis of Aeromonas hydrophila by inhibiting production of aerolysin and biofilm. Food Control.

[14] Nayak SK (2020). Current prospects and challenges in fish vaccine development in India with special reference to Aeromonas hydrophila vaccine. Fish Shellfish Immunol..

[15] Anjur N, Sabran SF, Daud HM, Othman NZ (2021). An update on the ornamental fish industry in Malaysia: Aeromonas hydrophila-associated disease and its treatment control. Vet. World.

[16] Mzula, A., Wambura, P. N., Mdegela, R. H. & Shirima, G. M. Current state of modern biotechnological-based Aeromonas hydrophila vaccines for aquaculture: A systematic review. *BioMed Res. Int.* **2019** (2019). 10.1155/2019/3768948 PMC6699303 31467887

[17] Cao Y (2020). Characterization and application of a novel Aeromonas bacteriophage as treatment for pathogenic Aeromonas hydrophila infection in rainbow trout. Aquaculture.

[18] Xu T (2023). A Global survey of hypervirulent aeromonas hydrophila (vAh) identified vAh strains in the lower mekong river basin and diverse opportunistic pathogens from farmed fish and other environmental sources. Microbiol. Spectrum.

[19] Dinçtürk E, Tanrıkul TT (2021). Yersinia ruckeri and Pseudomonas fluorescens co-infection in rainbow trout (Oncorhynchus mykiss Walbaum, 1792). Aquac. Res..

[20] Banerjee G, Ray AK (2017). The advancement of probiotics research and its application in fish farming industries. Res. Vet. Sci..

[21] Lv X (2018). A novel bacteriocin DY4-2 produced by *Lactobacillus plantarum* from cutlassfish and its application as bio-preservative for the control of Pseudomonas fluorescens in fresh turbot (Scophthalmus maximus) fillets. Food Control.

[22] Liu L, Chi H, Sun L (2015). Pseudomonas fluorescens: Identification of Fur-regulated proteins and evaluation of their contribution to pathogenesis. Dis. Aquatic Organ..

[23] Mahmoud MM, El-Lamie MM, Kilany OE, Dessouki AA (2018). Spirulina (Arthrospira platensis) supplementation improves growth performance, feed utilization, immune response, and relieves oxidative stress in Nile tilapia (Oreochromis niloticus) challenged with Pseudomonas fluorescens. Fish Shellfish Immunol..

[24] Fathy RM, Mahfouz AY (2021). Eco-friendly graphene oxide-based magnesium oxide nanocomposite synthesis using fungal fermented by-products and gamma rays for outstanding antimicrobial, antioxidant, and anticancer activities. J. Nanostruct. Chem..

[25] Kumar V, Roy S, Meena DK, Sarkar UK (2016). Application of probiotics in shrimp aquaculture: Importance, mechanisms of action, and methods of administration. Rev. Fisheries Sci. Aquac..

[26] Bondad‐Reantaso, M. G. *et al.* Review of alternatives to antibiotic use in aquaculture. *Reviews in Aquaculture* (2023).

[27] Ni M (2014). The physiological performance and immune responses of juvenile Amur sturgeon (Acipenser schrenckii) to stocking density and hypoxia stress. Fish Shellfish Immunol..

[28] Chen C-C (2019). Antimicrobial activity of Lactobacillus species against carbapenem-resistant Enterobacteriaceae. Front. Microbiol..

[29] Bove P (2012). Probiotic features of *Lactobacillus plantarum* mutant strains. Appl. Microbiol. Biotechnol..

[30] Tran, B. D. *et al.* Screening streptomycin resistant mutations from gamma ray irradiated *Bacillus subtilis* B5 for selection of potential mutants with high production of protease. *VNU J. Sci. Nat. Sci. Technol.* **32** (2016)

[31] Grangette C (2005). Enhanced antiinflammatory capacity of a *Lactobacillus plantarum* mutant synthesizing modified teichoic acids. Proceed. Nat. Acad. Sci..

[32] Tejada-Simon M, Lee J, Ustunol Z, Pestka J (1999). Ingestion of yogurt containing *Lactobacillus acidophilus* and *Bifidobacterium* to potentiate immunoglobulin A responses to cholera toxin in mice. J. Dairy Sci..

[33] Grangette C (2004). Enhanced mucosal delivery of antigen with cell wall mutants of lactic acid bacteria. Infect. Immun..

[34] Sorokulova IB (2008). The safety of two *Bacillus* probiotic strains for human use. Dig. Dis. Sci..

[35] Cutting SM (2011). *Bacillus* probiotics. Food Microbiol..

[36] Huang J-M, La Ragione RM, Nunez A, Cutting SM (2008). Immunostimulatory activity of *Bacillus* spores. FEMS Immunol. Med. Microbiol..

[37] Shelburne CE (2007). The spectrum of antimicrobial activity of the bacteriocin subtilosin A. J. Antimicrob. Chemother..

[38] Tsai C-C (2005). Antagonistic activity against Salmonella infection in vitro and in vivo for two *Lactobacillus* strains from swine and poultry. Int. J. Food Microbiol..

[39] Aly SM, Ahmed YA, Abdel-Aziz A (2008). acidophilus, as potential probiotics, on the immune response and resistance of Tilapia nilotica (Oreochromis niloticus) to challenge infections. Fish Shellfish Immunol..

[40] Azhdari SM, Pournaki SK, Tavabe KR, Hosseni SV, Bagheri D, Javanmardi S, Azhdari A, Frinsko M (2023). Effects of *Bacillus subtilis* and *Lactobacillus plantarum* probiotics on the Litopenaeus vannamei growth performance, hemolymph factors, and physicochemical parameters. Aquac. Rep..

[41] Xie, J., Zhang, R., Shang, C. & Guo, Y. Isolation and characterization of a bacteriocin produced by an isolated *Bacillus subtilis* LFB112 that exhibits antimicrobial activity against domestic animal pathogens. *Afr. J. Biotechnol.* **8** (2009)

[42] Dawood MA, Koshio S, Abdel-Daim MM, Van Doan H (2019). Probiotic application for sustainable aquaculture. Rev. Aquac..

[43] Mohapatra S (2012). Use of different microbial probiotics in the diet of rohu, Labeo rohita fingerlings: Effects on growth, nutrient digestibility and retention, digestive enzyme activities and intestinal microflora. Aquac. Nutr..

[44] Hidalgo M, Skalli A, Abellán E, Arizcun M, Cardenete G (2006). Dietary intake of probiotics and maslinic acid in juvenile dentex (Dentex dentex L.): effects on growth performance, survival and liver proteolytic activities. Aquac. Nutr..

[45] Merrifield D, Bradley G, Baker R, Davies S (2010). Probiotic applications for rainbow trout (Oncorhynchus mykiss Walbaum) II. Effects on growth performance, feed utilization, intestinal microbiota and related health criteria postantibiotic treatment. Aquac. Nutr..

[46] Nandi A, Banerjee G, Dan SK, Ghosh K, Ray AK (2017). Probiotic efficiency of Bacillus sp. in Labeo rohita challenged by Aeromonas hydrophila: assessment of stress profile, haemato-biochemical parameters and immune responses. Aquac. Res..

[47] Kheadr E, Dabour N, Le Lay C, Lacroix C, Fliss I (2007). Antibiotic susceptibility profile of bifidobacteria as affected by oxgall, acid, and hydrogen peroxide stress. Antimicrob. Agents Chemother..

[48] Tesfaye O, Muleta D, Desalegn A (2022). In vitro antimicrobial properties of apis mellifera L. and Meliponulla beccarii L. honeys from Kellem and West Wollega Zones. Western Ethiopia. Int. J. Food Prop..

[49] Han B (2022). Effects of acute hypoxic stress on physiological and hepatic metabolic responses of triploid rainbow trout (Oncorhynchus mykiss). Front. Physiol..

[50] Raisi A (2020). Evaluation of the anesthetic and tranquilizing effects of clove powder (Syzygium aromaticum) and lavender oil (Lavandula officinalis) in convict cichlid fish (Cichlasoma nigrofasciata). Iran. J. Vet. Surg..

[51] Hoseini SM, Ghelichpour M (2012). Efficacy of clove solution on blood sampling and hematological study in Beluga, Huso huso (L.). Fish Physiol. Biochem..

[52] Harikrishnan R, Rani MN, Balasundaram C (2003). Hematological and biochemical parameters in common carp, Cyprinus carpio, following herbal treatment for Aeromonas hydrophila infection. Aquaculture.

[53] Drabkin DL (1945). Hemoglobin, glucose, oxygen and water in the erythrocyte: a concept of biological magnitudes, based upon molecular dimensions. Science.

[54] Nonaka M (1984). Purification of a major serum protein of rainbow trout (Salmo gairdneri) homologous to the third component of mammalian complement. J. Biol. Chem..

[55] Budiño B, Cal RM, Piazzon MC, Lamas J (2006). The activity of several components of the innate immune system in diploid and triploid turbot. Comp. Biochem. Physiol. Part A Mol. Integr. Physiol..

[56] Yano, T. Assay of hemolytic complement activity. *Tech. Fish Immunol.*, 131-141 (1992)

[57] Siwicki AK, Anderson DP, Rumsey GL (1994). Dietary intake of immunostimulants by rainbow trout affects non-specific immunity and protection against furunculosis. Vet. Immunol. Immunopathol..

[58] Rahimi S (2020). Co-culturing *Bacillus subtilis* and wastewater microbial community in a bio-electrochemical system enhances denitrification and butyrate formation. Chem. Eng. J..

[59] Chen YS, Yanagida F, Shinohara T (2005). Isolation and identification of lactic acid bacteria from soil using an enrichment procedure. Lett. Appl. Microbiol..

