# Supplementary material for: Probiotic performance of B. subtilis MS. 45 improves aquaculture of rainbow trout Oncorhynchus mykiss during acute hypoxia stress
Source: Sci Rep. 2024 Feb 14;14:3720. doi: 10.1038/s41598-024-54380-7 (PMC10866961; doi:10.1038/s41598-024-54380-7)
Supplement: Supplementary file 1 — Supplementary Table 1. [file 41598_2024_54380_MOESM1_ESM.docx]

**Supplementary Table 1.** Primers used for gene expression analysis in *Oncorhynchus mykiss* in normoxia and hypoxia conditions

| Gene | Primer (5'to 3') | Accession number |
| --- | --- | --- |
| HIF-1α | TCTGAGGACGGGGACATGAT | AF304864.1 |
|  | GGTCTGAGCAGTGGAGAACC |  |
| HIF-2α | GGTTACATCAGACGGCGACA | XM_021576379.1 |
|  | CCTTCTTCCCAGTGCCATTTT |  |
| FIH1 | ACAGCCCTATCTGGAACGACTC | NM_001281328.1 |
|  | CCACTGGTTGCTCGTTGTTTAT |  |
| IFN-γ | TACCCTCACCTTCCCACCA | NM_001124620.1 |
|  | TTCCTGCGGTTGTCCTTCTT |  |
| TNF-α | GGCGAGCATACCACTCCTCTGA | AJ401377.1 |
|  | AGCTGGAACACTGCACCAAGGT |  |
| IL-1β | ACGGTTCGCTTCCTCTTCTACA | AJ557021.2 |
|  | GCTCCAGTGAGGTGCTGATGAA |  |
| IL-8 | GTCAGCCAGCCTTGTCGTTGT | NM_001124362.1 |
|  | CGTCTGCTTTCCGTCTCAATGC |  |
|  |  |  |
| β-actin | ATGGGCCAGAAAGACAGCTACGTG  CTTCTCCATGTCGTCCCAGTTGGT | NM_001124235.1 |
